# Cognition in elite soccer players: a general model

**DOI:** 10.3389/fpsyg.2024.1477262

**Published:** 2024-12-11

**Authors:** Thomas Habekost, Jacob Ovesen, Jes Buster Madsen

**Affiliations:** ^1^Department of Psychology, University of Copenhagen, Copenhagen, Denmark; ^2^F. C. Copenhagen, Copenhagen, Denmark; ^3^Saudi Pro League, Riyadh, Saudi Arabia

**Keywords:** soccer (football), cognition, perception-action cycle, model, review

## Abstract

This paper presents a general model of the cognitive processes involved in each play situation of soccer at the elite level. Theoretically the model draws on general frameworks from cognitive psychology and neuroscience, in particular the affordance competition hypothesis and the reward prediction error theory. The model includes three functional stages: situational assessment, action selection and execution, and outcome assessment. The three stages form a perception-action cycle that corresponds to a single play situation. The cognitive processes operating at each functional stage are described and related to soccer research by a review of 52 empirical studies. The review covers the main cognitive processes that have been studied in soccer research: visual orientation and attention, pattern recognition, anticipation, working memory, action selection and decision making, executive control processes, as well as behavioral and cognitive learning. The model accommodates the wide variety of findings in the empirical literature and provides a general organizing frame for cognitive soccer research at the elite level. The influence of emotional and stress-related factors on cognition are also discussed. Four general limitations of the existing soccer research are identified, and suggestions for future studies include development of more naturalistic and interventional study designs. By specifying the different cognitive processes in soccer and their dynamic interactions the model has many applied perspectives for soccer training at the professional level. Overall, the paper presents the first integrated process model of cognition in elite soccer players with implications for both research and practice.

## 1 Introduction

Soccer is the most popular sport in the world: hundreds of millions play the game at amateur level, and even more follow professional teams as they compete in national leagues and international tournaments. In this multi-billion dollar industry soccer clubs devote enormous resources to optimize the performance of their players. The efforts have traditionally focused on tactical, technical, and physiological efficiency, but there is a growing area of research investigating cognitive processes involved in elite soccer performance (Scharfen and Memmert, 2019). This interest is well motivated given the nature of the game. Soccer players perform in a fast-paced, dynamically changing environment, where the simultaneous actions of many opponents and teammates must be rapidly perceived and predicted in order to respond effectively at any given moment. This challenging task engages a wide variety of cognitive processes such as visual perception, attention, anticipation, working memory, social cognition, and executive control, which must work flexibly together to sustain the player’s actions.

In the last two decades many aspects of cognition in soccer have been investigated empirically, as detailed later in this paper. The studies have employed a mixture of general cognitive testing and soccer-related experimental tasks, typically contrasting the performance of elite and less skilled players, and in some cases correlating these measures to performance on the pitch. Many interesting findings have been made, but studies have typically focused on isolated aspects of cognition without reference to a general theoretical framework. Broader theoretical conceptualizations of cognition in soccer have been put forward, in particular relating to the decision making aspect (Petiot et al., 2021; Raab, 2012). However so far no theory has encompassed the full spectrum of cognitive processes involved in soccer. Given the complexity of cognitive mechanisms that are involved in soccer, especially their dynamic interactions, an integrative process model could have large utility for the field. The holistic perspective provided by such a model could also guide future research by pointing to aspects of cognition in soccer that have not received empirical investigation so far. Not least, from a practical point of view, a detailed analytical framework could be highly useful to devise targeted cognitive training for soccer players and to open the possibility for cognition-based talent identification.

In this article we present a general model of cognitive processes in elite soccer players and review how the model relates to the current empirical evidence. The focus on soccer at the elite level is in line with most research in the field, and also reflects the fact that cognitive processes may be qualitatively different between novices and elite athletes, for example with respect to automaticity and the involvement of conscious thinking (Starkes et al., 2004). We discuss strengths and limitations of the model, offer a critical assessment of the empirical research in the field and, based on this, suggest new directions for scientific investigations of cognitive processes in elite soccer. We also outline applied perspectives of the model in relation to professional soccer training.

## 2 A model of cognitive processes in elite soccer

### 2.1 General principles of the model

**Figure 1:**
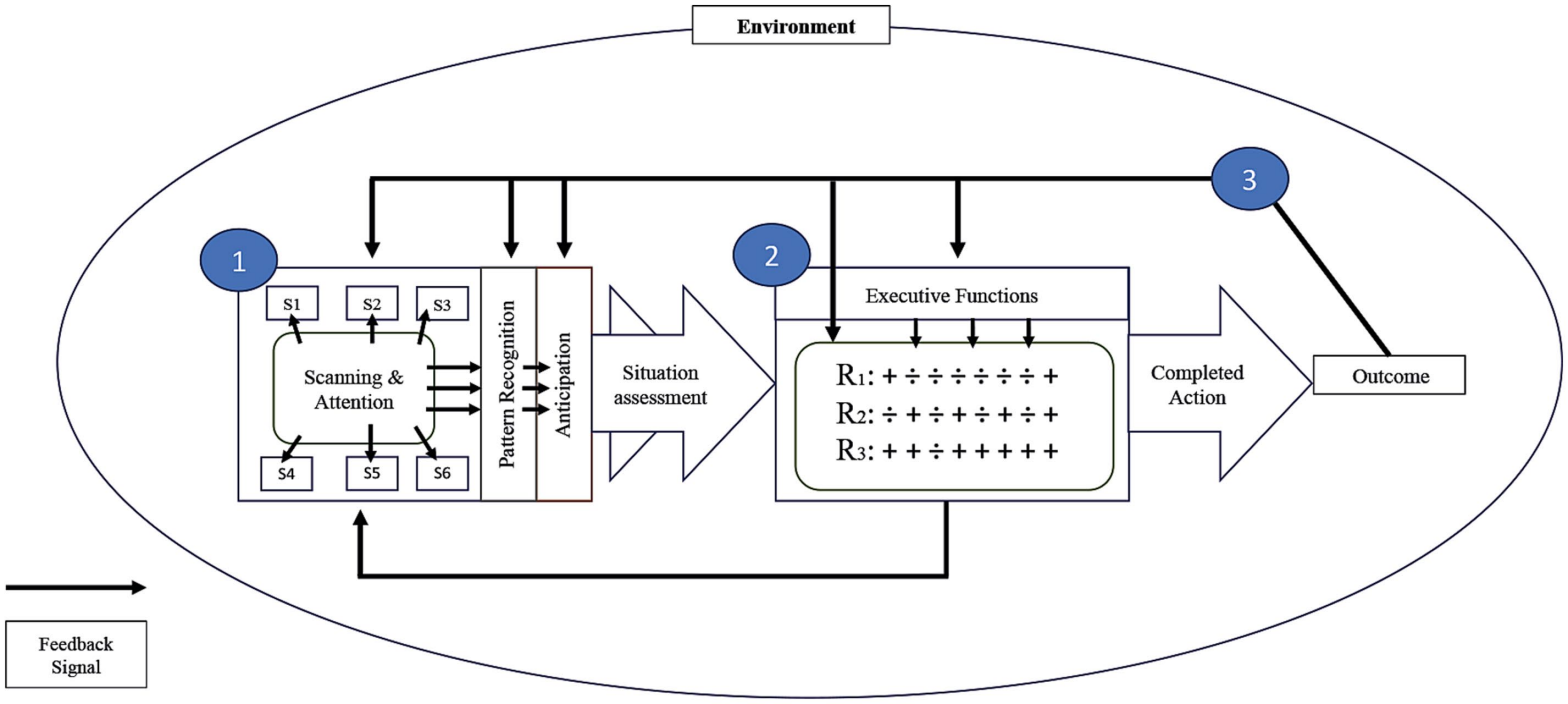
The three stages of a perception-action cycle in soccer. Stage 1, assessment of the current play situation; Stage 2, action selection and execution; Stage 3, assessment of outcome and systemic feedback. S_1-6_, surrounding stimuli; R_1-3_, competing response options. Plusses and minuses indicate excitatory and inhibitory influences on response selection.

The model depicted in Figure 1 describes the relation between the cognitive processes involved in each individual play situation of soccer at the elite level. The model describes the basic situational unit in the form of a single perception-action cycle. We define the basic situational unit as the events related to a single tactical action in soccer. If the player possesses the ball, an action may be a pass, a shot at the goal, or a dribble. If the player does not possess the ball, an action can be an attempt to tackle, to block the ball or the running course of an opponent player, or to move into a different part of the playing field for defensive or attacking purposes. The model applies both to situations with and without ball possession, where the latter is far more frequent. The typical duration of a single play situation is a few seconds, which allows for some perceptual updating and response preparation within the situation. Thus, the concept of an individual play situation and its corresponding action is defined at the tactical level, which is a more complex type of behavior than individual movements (see Schmidt and Lee, 2019, for research on the latter type of processes).

The model describes the cognitive processes during the play situation at different levels of analysis. At the most general level, the model describes how the player goes through three functional stages from start to end of the cycle: (a) assessment of the current play situation, (b) action selection and execution, and (c) assessment of outcome and systemic feedback. The model works in a cascading way, so earlier stages continue to be active during the processing at later stages and can influence these until an action has been fully executed. This way, Stage 1 continues to deliver information about the current play situation during the processes at Stage 2, which can for example lead to inhibition of an action if circumstances change midway. There are also feedback connections between Stage 2 and 1, so the process of action selection can initiate new exploratory behavior before an action is decided upon and fully executed. Several of the processes within stages also occur in parallel, for example the selection between response options at Stage 2. Therefore, although the model as a whole has a serial processing character, it encompasses parallel and interactive processes both within and between the three stages. Each functional stage depends on specific cognitive processes, as described in the following sections.

### 2.2 The three stages of a perception-action cycle in elite soccer

#### 2.2.1 Stage 1

The function of the first stage is to provide the player with a continuous assessment of the current play situation. This mainly depends on visual perception, but also on other sensory modalities such as audition, somatosensation and proprioception. The perceptual processes are intimately connected to attentional functions, which direct focus toward particular aspects of the situation. The attentional focus is supported and elaborated by active exploratory movements, in particular visual orienting, and relies heavily on previous learning. Much of the orienting processes occur at an automatic cognitive level, but conscious intentions can also influence the player’s behavior via executive control processes. Soccer-specific pattern recognition, for example of particular configurations of the surrounding players in relation to the ball, is also a central aspect of this stage. In addition, anticipations of the actions of other players are an integral part of the situation assessment, for example based on the postural positions of teammates and opponents. The outcome of Stage 1 is a dynamically updated assessment of the current play situation, including anticipations of the immediate future, which is represented in the player’s working memory.

#### 2.2.2 Stage 2

The function of the second stage is to evaluate the currently relevant response options in order to select and execute a particular action. The response options are heavily narrowed down by the situational assessment from Stage 1, which activates only a few responses in the player’s procedural long-term memory. The response options are simultaneously activated in specific neural populations within motor-related areas of the brain and compete for action selection. The activation of response options is not purely cerebral, but also have effects in the body in the form of muscle activations and preliminary movements. For a defensive player holding the ball, the relevant alternatives could for example be to pass the ball back to the goal keeper, to direct a long pass to a teammate further up the field, or to make a short pass to a fellow defensive player. Due to the speed of the game the selection and execution of actions largely occurs at an automatic, non-conscious level. The activation of specific response options depends on previous learning, which in the case of elite players amounts to thousands of hours of systematic training in specific play situations. By way of priming mechanisms, the response activations are also influenced by recent events in the game, for example following a successful encounter with an opponent player or a missed shot at the goal. The action selection is determined by an implicit evaluation of the potential risks and benefits related to each potential action, which also includes the probability of carrying the action out as intended. The evaluation is implicit in the sense that it depends on excitatory and inhibitory activity (formed by previous learning) in neural networks representing the competing responses, rather than a conscious deliberation of choice options. The level of risk-taking in the decision process depends on personal characteristics of the player, including the current level of self-confidence, as well as the overall game situation. While the action selection process itself is essentially non-conscious, executive processes can influence the outcome for example by representing team strategy and other conscious intentions. The automaticity of the response selection varies both between situations and individuals. A player may be trained to systematically carry out a specific action in a particular situation, but can also be trained (or personally inclined) to act in a more flexible or creative manner. A largely automatic response mode corresponds to a strong activation of just one response in Stage 2, whereas a more flexible response mode entails significant competition between several response options and typically more feedback interaction with the information gathering processes of Stage 1. In any case, the outcome of Stage 2 is the full execution of a particular action. The action implies an expectation of its likely outcome, based on previous learning, which feeds into the next stage of the perception-action cycle.

#### 2.2.3 Stage 3

After the action has been carried out the player perceives the outcome. The perception of the outcome is directly coupled to the player’s intention for the action and implies an assessment of its successfulness. The assessment activates the brain’s reward systems and makes the player either more or less likely to repeat the same action in similar future situations. The degree of behavioral modification depends on the difference between the expected and actual outcome of the action, so large discrepancies (reward prediction errors) will lead to more changes. Specifically, the feedback is implemented via modifications of the cognitive and neural settings that are involved in Stages 1 and 2, for example leading to changes in the orientation behavior at Stage 1 or different response tendencies at Stage 2. This way, Stage 3 feeds back into new cycles of perception and action, provides opportunities for learning, and modifies the perceptual processing, decision making, and motor execution in future play situations.

## 3 Empirical research on cognitive processes in soccer

Empirical research on cognitive processes in soccer was pioneered in the 1990s (Williams et al., 1994; Williams and Davids, 1998) and has developed much during the first two decades of the 21st century. In this section we present a narrative review of this research field, theoretically organized by the functional categories and cognitive processes described in our model. In each subsection we describe the main empirical findings related to the cognitive process in question, discuss methodological and conceptual issues, and give an assessment of the current state of the evidence. The studies included in the narrative review were selected by the following criteria: they should report on empirical investigations of cognitive processes in soccer, include elite or highly experienced players, and be published in English language peer-reviewed scientific journals in 2003 or later. Studies were not included if the investigation was mainly focused on clinical issues (e.g., concussion) or stress/fatigue, or if other sports were the main focus of the investigation. To avoid the added complexity of developmental processes, studies were also not included if participants were on average under the age of 18. The studies were found through searches in Web of Science by combinations of selected key words (e.g.: “soccer” or” football” and “cognition,” “scanning,” “anticipation,” or “decision making”) combined with exclusion criteria (e.g.: “not concussion,” “not injury,” and “not youth”). This was followed by screening of abstracts and final evaluation of the content of the studies. Additional studies were found through references in the selected articles. In total, 52 empirical studies are included in the narrative review. In addition, a number of more broadly focused theoretical and empirical papers are included to provide general context and background for the different subsections.

### 3.1 Stage 1: assessment of the current play situation

#### 3.1.1 Visual orientation and attention

Soccer is a highly dynamic sport and to perform effectively players must continuously update their perception of the current situation. The most important source of perceptual information in soccer is vision, and relevant visual stimuli typically surround the player in all directions. Therefore effective orientation behavior in the form of eye movements, head turns, and whole body movements is presumably crucial to perform well. For this reason, and perhaps also because orientation behavior is readily observable, this has been one of the most active areas of investigation within cognitive soccer research, as well as in other sport disciplines (Klostermann and Moeinirad, 2020; Silva et al., 2022). It is also an area of high interest to coaches (Pulling et al., 2018). Orientation behavior is intimately linked to attentional functions, which guide exploratory movements to ensure that the most relevant information is focused on and perceived. In soccer visual attention is typically directed at the ball, the positions of opponents and teammates, open and closed spaces on the field, or postural cues for anticipating the actions of other players. General attentional priorities such as these guide the player’s orientation behavior, but how they are weighted is often determined by specific events that occur during the visual exploration. This way, the relation between orientation behavior and attention is highly interactive. Conscious intentions, for example specific instructions by the coach, may also influence orienting behavior by way of executive control processes; however this top-down aspect has not been addressed directly in the empirical literature.

The large majority of studies on visual orientation behavior in soccer has focused on eye movement patterns (McGuckian et al., 2018b). The empirical findings in this research field are mixed, and there seems to be no general difference between elite and amateur players with regard to the frequency of eye movements and the duration of fixations. For example, Cañal-Bruland et al. (2011) found that expert soccer players made fewer fixations of longer duration than controls when looking at images of play situations, whereas Roca et al. (2011) found the opposite pattern. Rather than showing general characteristics of eye movements in skilled players, many studies suggest that a number of variables modify scanning rates and fixation patterns in different play situations. A commonly studied situation is ball reception, often in connection with subsequent passing. Natsuhara et al. (2020); see also Oppici et al. (2017) studied how soccer players reacted to life-sized videos of real play scenes, where a physical ball was ejected toward the participant according to the play situation. This was done to simulate ball reception and passing decisions. Natsuhara et al. (2020) found that skilled players fixated more than controls on opponents before receiving, and more on non-marked attackers and the team-mate to receive the pass before passing. This suggests that skilled players search the visual surroundings for more relevant information than controls, flexibly adapted to the current play situation. Similar findings have been made in studies of visual orientation behavior as a function of the player’s distance to the ball. Roca et al. (2013) found that skilled players typically made shorter and more numerous fixations when the ball was far away, presumably to perceive the general pattern of play. On the other hand, when the ball was close, skilled players made fewer fixations of longer duration and focused mainly on postural cues of the player in ball possession, presumably to predict the next movement. In contrast, less skilled players tended to focus directly on the ball in both types of situations, indicating a lack of dynamic adaptation of their visual exploration behavior.

Another line of studies has focused on the influence of individual characteristics. Roca et al. (2018) investigated how individual differences in creativity are related to visual search strategies. In this study, participants were presented with life-sized video simulations of attacking situations while in ball possession. The most creative players (i.e., those who created more flexible and original decisions) differed in visual search strategy by making more short fixations of informative locations. Roca et al. interpreted this as reflecting a broader attentional focus in creative players. Individual differences in working memory capacity (see also Section 3.1.4) might also be relevant for visual orientation behavior. However, Harris et al. (2020) found that gaze strategy did not differ between two groups of athletes (where one group included soccer players) with different working memory capacity, as measured by multiple object tracking (see also Cañal-Bruland et al., 2011).

Another important question concerns the type of stimuli that elite players typically attend to. This is often studied in relation to anticipation processes (see also Section 3.1.3). There is evidence that superior perceptual abilities in sports lead to better anticipation (Brams et al., 2019; Williams and Jackson, 2019) and that more skilled players, who tend to anticipate better, also employ a distinct visual search strategy. Whereas experts and novices alike will often fixate on the ball and the player in possession of it, which aligns with a primary reliance on postural cues, experts will do this significantly less (Casanova et al., 2013; Roca et al., 2011), indicating their superior integration of other sources of information. Indeed, when provided with contextual priors in the form of opponents’ action tendencies, experts but not novices spend significantly more time watching other elements in the visual field than the player in possession of the ball (Gredin et al., 2018). Additionally, when the main action is far away, more skilled players will focus relatively less on the player in possession of the ball and more on other surrounding subjects (Roca et al., 2013). These results further highlight that elite soccer players incorporate multiple sources of information when anticipating and can adjust their strategy depending on the quality of available information.

Most research on visual orientation in soccer is laboratory-based, which does not necessarily generalize to real-life soccer play. Gaze patterns may be different for artificial, two-dimensional stimuli compared to immersion in real-life environments (Dicks et al., 2010; Foulsham et al., 2011), and virtual reality environments may also elicit different exploration behavior than natural situations (Pastel et al., 2021). To address this issue, a few recent studies have measured visual orientation behavior during real-life games and related it directly to performance on the pitch. McGuckian et al. (2018a) found that the frequency of head turns in live matches were related to faster passing response time. The study did not find a relation between orientation behavior and the success rate of the passes, but this has been demonstrated in more recent studies. Jordet et al. (2020); see also Phatak and Gruber (2019) studied orientation behavior in the form of head turns away from the ball, which they video recorded during 21 competitive matches in a group of players from the English Premier League. Jordet et al. (2020) found positional differences in scanning behavior: central midfielders and central defenders scanned more, and forwards least. They also found differences related to the game situation, for example with less scanning under tight opponent pressure or close to the opponent goal. Importantly, Jordet et al. (2020) found that the probability of making successful passes increased with scanning frequency. However the effects were not large, and the authors concluded that the frequency of scanning (head turns) seems to have only a small, yet positive role in elite players’ performance. Based on videos of 72 professional players in live matches, Caso et al. (2023) studied head and body movements (Visual Exploratory Activities; VEAs) prior to receiving the ball. The players belonged to the same club, which used a 3-man passing system: the final pass would be directed at the player being measured, whereas the penultimate pass represented valuable early information for this player. Caso et al. (2023) found that the amount of VEAs in relation to the penultimate pass predicted the adequacy of the subsequent pass by the player. Caso et al. (2023) also found effects of playing position, where midfielders made more VEAs than defenders and attackers. Aksum et al. (2020) studied eye movements in 5 elite players during match play in the Norwegian premier league. Eye movement patterns varied with attacking-defending phases, as well as the complexity of the situation: fixations were longer when a higher number of “areas of interest” (e.g., ball, teammate, opponent) were present in the visual field, suggesting that longer processing time was needed when more information was available. Notably, Aksum et al. (2021) also found that scan times were significantly shorter in real-life play than typically reported for laboratory tasks, which questions the validity of such visual orientation studies in soccer. This finding is in line with the review of McGuckian et al. (2018b), who also found that eye movement patterns varied with the representativeness of the study design [see also Aksum et al. (2021)].

In summary, research has shown that visual orientation behavior and related attentional processes vary systematically with the play situation, as well as with individual player characteristics such as creativity. Skilled players tend to focus their attention on the most informative aspects of the situation, which can vary on several dimensions. Whereas the majority of studies in this field are laboratory-based, a few recent studies report on relations between real-life orientation behavior and game performance, especially passing, but this type of evidence is still limited. Another limitation of research on visual orientation and attention, which also applies to cognitive soccer research in general, is that studies typically focus on situations with ball possession or reception, but not the large majority of play situations where the player in not in immediate contact with the ball. Also, a lack of intervention studies makes it difficult to know if and how orientation behavior is causally related to higher performance, or merely correlated with it. The executive control aspect of orienting in soccer is neither addressed in current research. Thus, in spite of the relatively large research interest in this subfield, many questions remain unanswered.

#### 3.1.2 Pattern recognition

There is no clear evidence that basic perceptual functions are superior in elite athletes (Ward and Williams, 2003), but specialized pattern recognition abilities have long been considered central for development of expertise (Chase and Simon, 1973). Studies of expertise in numerous fields have shown that highly specific knowledge structures that represent relations between individual items (e.g., “templates”; Gobet and Simon, 1996) develop after extensive practice in a particular domain. Williams et al. (2006) presented a method for studying pattern recognition in soccer by using point-like representations of play situations. For highly skilled players they found no difference in memory performance for video clips of play situations with these abstract displays and fully detailed videos, but for less skilled participants there was a significant degradation. This indicates that experts are better able to pick up abstract structural play configurations, whereas less skilled players rely on more superficial sensory features. In a follow-up study Williams et al. (2012) found that dynamical motion aspects of the patterns, not static configurations, differentiated soccer experts from lesser-skilled observers. They also found that relative, not absolute, motion patterns are crucial. Other studies have further investigated which features of a play configuration is typically focused on by soccer experts. One finding is that centrally located attacking players seem to be especially critical for experts (North et al., 2009). This was indicated by the number of eye movements directed at these players as well as by experimental removal of player stimuli, which shows that movements of central attacking players hold essential information (North et al., 2017). However the experimental testing in these studies was based on general views of the soccer field, whereas in actual play the most important features are likely to vary with the position and functional role of each player. Even for a given position, there are presumably a great variety of playing patterns that are relevant to expert performance in soccer. Apart from the few general principles of pattern recognition in soccer that have been uncovered so far, the detailed nature of these patterns has not been characterized empirically. In addition, the link between pattern recognition skills and actual soccer performance has not been established.

Another important aspect of pattern recognition is the relation to anticipatory processes. From a theoretical perspective pattern recognition should be highly relevant to anticipation, since the identification of familiar situations and knowledge of their usual outcomes can be used to predict events in the present (Navia et al., 2018). Moderate correlations between proficiencies in these two functions have been documented, and eye tracking data indicate visual search strategies that show both similarities and differences when performing anticipation tasks and recognition tasks (North et al., 2009). Also, participants tend to report on the basis of more sophisticated memory representations when anticipating than when recognizing (North et al., 2016). Thus, although there is likely some overlap between pattern recognition and anticipation, the processes appear to be functionally distinct and are represented separately in our model.

#### 3.1.3 Anticipation

Interceptive strategic sports like soccer are defined by a fast-paced and dynamic environment in which actions must be executed quickly to take advantage of opportunities before they disappear. As such, the ability to predict future events and prepare actions ahead of their occurrence is advantageous (Navia et al., 2018). There are multiple definitions of anticipation in the sports literature, but for most soccer-related studies, anticipation is primarily conceptualized as the ability to predict the actions of other players, usually opponents (Zhao et al., 2022). Anticipations of the movements and future positions of objects in motion have also been investigated (Craig et al., 2009), but these studies make up only a fraction of the literature and will not be discussed here. Anticipation has been highlighted as a key factor in successful performance in many different team sports (Ashford et al., 2021), and research generally supports the notion that elite athletes in strategic sports possess superior anticipatory abilities within their domains of expertise (Petiot et al., 2021). In soccer, coaches from Brazil to Germany suggest its importance in different facets of decision making (Klatt et al., 2019).

In our model anticipation is considered a part of Stage 1 and relates to predictions about the actions of other players and the immediate development of the current play situation. Predictions about the consequences of one’s own actions are regarded as part of Stage 2 in our model (see section 3.2). Likely based on more complex cognitive operations than simple perception, in particular advanced pattern recognition processes, anticipation serves to provide accurate predictions of how the situation might change in the next few seconds. Soccer-related anticipation studies can be classified depending on the kinds of information and cognitive strategies participants are thought to employ when performing tasks. Most studies assume that soccer players rely on postural cues to predict the actions of opponents. Indeed, skilled soccer players seem to possess a superior ability to quickly identify the direction of biological motion, not just for soccer-related motions, but also for more general kinds of human kinematics (Romeas and Faubert, 2015). Typically, anticipation studies require participants to watch a clip from a real soccer match in which an opposing player in possession of the ball is about to take an action. The clip is occluded before the action and participants must predict what the action is going to be. Actions are considered successfully anticipated if participants’ predictions correspond to the action taken by the opponent in the clip. Generally, studies support the notion that skilled soccer-players outperform less skilled players on such postural tasks (Casanova et al., 2013; North et al., 2016; Roca et al., 2013, 2011). Many studies do not include measures of response time, but those that do generally show that more skilled players anticipate significantly quicker (Gredin et al., 2018). Further, more skilled soccer players are better at anticipating deceptive moves (Wright et al., 2013), at generating relevant action options for an opposing player (Belling et al., 2015), and at generating more verbal statements of higher complexity about their own thought processes during anticipation (Roca et al., 2013, 2011). At the neurophysiological level, these processes may be supported by mirror neuron systems (Gorgan Mohammadi and Ganjtabesh, 2024).

It seems clear that elite soccer players possess a superior ability to anticipate the actions of opponents, but they may utilize more than just postural cues to do so. Several studies supply participants with other helpful information such as the opponent’s action tendencies or structural knowledge about players’ positions on the field. Gredin et al. (2018) found that the provision of contextual priors through knowledge about opponents’ action tendencies benefitted anticipation for both novice and expert soccer-players, but when opponents acted against their tendency, a detrimental effect was only observed for novices. This suggests that experts may be more skilled at determining when contextual information should be relied on or not. Similarly, Thomas et al. (2022) found that both skilled and less skilled players could covertly learn and benefit from opponents’ action tendencies when anticipating, but when tendencies were suddenly switched, only skilled players adapted their expectations. Knowledge of the action tendencies of teammates is also important for anticipation, and developing this shared knowledge is a major focus in collective training sessions [see, e.g., Blaser and Seiler (2019)].

By varying the reliability of postural cues (by changing the time of clip-occlusion and the distance to the opposing player) and contextual information (by varying the consistency of action-tendencies), researchers have found that expert soccer players employ Bayesian probability-based strategies when anticipating (Gredin et al., 2021). Generally, skilled soccer players seem to possess a greater advantage in postural anticipation (North et al., 2016), and if postural cues are reliable, they will primarily base their anticipatory judgment on this source of information (Gredin et al., 2021). Further, though they may initially rely on contextual information, experts will switch to postural cues right before the opponent’s action execution, when kinematic information is most reliable. A similar switch is not observed in novices (Gredin et al., 2018). However, when kinematic information is unreliable due to a greater distance from the action, early occlusion, or other kinds of image manipulation, contextual priors of both low and high reliability as well as structural information take precedence in anticipation (Gredin et al., 2018; North et al., 2016). Taken together, these findings show that expert soccer players utilize multiple different sources of information when anticipating, and that their superior performance may especially be due to an ability to adapt their anticipation strategy depending on the relative reliability of the different information sources.

As has been highlighted above, most studies make inter-group comparisons, showing differences in anticipation between groups of different skill levels. Very few studies, however, make intra-group comparisons between players at the same skill level. Because groups typically vary on several other variables than skill level such as years of experience, hours trained per week, playing position, and belief in own ability, it is difficult to determine if anticipation is linked directly to soccer performance or simply to greater familiarity with the game. Additionally it is difficult to determine to which extent anticipation is directly useful during soccer play or if more skilled players simply develop this ability without benefitting from it during matches. Intervention studies, in which anticipation processes are trained or experimentally manipulated, can potentially address this question, but limited research has been done in this area. In a review of video-based intervention studies of anticipation, Zhao et al. (2022) found that anticipation could be trained by such interventions. Additionally, in the single study that included transfer tests to soccer performance, anticipation improvements seemed to benefit performance (Gabbett and Mulvey, 2008). However, due to limited and conflicting findings on this question, it remains unclear how and to which extent anticipation is beneficial to actual soccer performance.

In a theoretical context, an additional issue can be raised regarding anticipation. It can be argued that the line between reaction and anticipation is blurred. “Reaction” seems to imply a response to an already unfolding event, whereas “anticipation” suggests the prediction of an event that may only occur with some probability. However issues arise when defining what perceptual features constitutes the event itself, and it may be impossible to determine when an event is absolutely certain to occur, meaning that every reaction could contain some element of anticipation. Conversely, anticipating athletes may simply have learned to “react” to cues that appear ambiguous to the untrained observer, but almost always precede a certain outcome. Biomechanical studies suggest that visual information becomes more reliable the closer it is to the execution of the action to be anticipated (Navia et al., 2018). As mentioned before, elite soccer players also tend to rely more on postural cues right before action execution (Gredin et al., 2018) and this very late perceptual information has proven especially useful when the opponent makes a deceptive move (Wright et al., 2013). If expert soccer players wait to anticipate until right before the opponent’s action execution, are they truly anticipating or simply reacting when the opponent can no longer inhibit a certain response? To support this latter notion, research indicates that better goalkeepers rely on reaction rather than anticipation when defending against a shot at the goal (Navia et al., 2018). Thus, there seems to be a tradeoff between speed (anticipation) and accuracy (reaction). Anticipation may allow a player to respond to an opponent’s action quicker, but the response may prove ineffectual if perceptual information is unreliable. Reacting may allow a player to respond to an opponent’s action more accurately, but the response may come too late to be effective. Ultimately, anticipation may be detrimental or beneficial depending on the situation. While for some actions it may be better to wait and react, others may occur so quickly that a player must rely on anticipation instead.

Overall, there is strong evidence that skilled soccer players are better at anticipating the behavior of other players than less skilled players. When anticipating, skilled players seem to utilize both postural cues, contextual information, and strategic knowledge of patterns. However, due to a lack of intervention studies and studies that compare individuals of a similar skill level, it remains uncertain to which extent anticipatory ability enhances soccer performance. Just as elite players utilize different kinds of information in an adaptive manner when anticipating, anticipation itself might be most useful when it is relied upon flexibly depending on the circumstances. When the actions of opponents and teammates occur too quickly to allow for a reaction, anticipation can be a useful way to create an understanding of the current play situation and thus allow for earlier initiation of the next phase of the perception-action cycle: action selection.

#### 3.1.4 Working memory

Given its central role in retaining and manipulating consciously available information, working memory influences several stages of the perception-action cycle. Chiefly, working memory serves as the store for the information that makes up the situational assessment of Stage 1. Through working memory the assessment is carried over to Stage 2, where it activates representations in procedural long-term memory (see Section 3.2.1). Within Stage 2 working memory is thought to play a different role, as it contributes to conscious executive processes that bias the action selection process. In this section we focus on the functions of working memory related to Stage 1, whereas Stage 2 functions are described in Section 3.2.2.

The relationship between working memory and soccer has been investigated extensively. Several studies have compared performance on working memory tasks to measures of soccer performance. Working memory capacity is typically measured with classical cognitive tests such as varieties of the operation span task, which require participants to retain and manipulate multiple pieces of information, or via multiple object tracking tasks. Studies have shown a link between multiple object tracking ability and more successful passes (Romeas et al., 2016). Further, soccer players tend to exhibit better executive functioning, including working memory, than athletes from non-strategic sports (Yongtawee et al., 2022) as well as a higher workload capacity during fast decision-making (Wang et al., 2020). Vestberg et al. (2012) have shown that Swedish national team soccer players outperformed lower ranking professional players on the design fluency test, a test of working memory and executive functioning, and in another study, researchers found that a design fluency score could substantially account for a player’s coach-rated soccer ability (Vestberg et al., 2020). On the other hand, one study found no relationship between working memory and creative decision-making on a computerized soccer-related decision-making task (Furley and Memmert, 2015), and another study failed to find differences in working memory abilities between soccer players of different skill levels (Glavas et al., 2023). Both studies used tasks specifically designed to measure working memory rather than a broader array of executive functions, and as such, their negative results may cast doubt on a selective positive influence of working memory on soccer performance. Taken together, results from this first line of studies are inconsistent and significant effects may depend on how broad a test of working memory is used as well as the choice of outcome measure.

Another category of research investigates the relationship between working memory and soccer performance by overloading working memory with irrelevant information or distractor tasks to measure the impact on simultaneous performance of a soccer related task. This line of studies is in part inspired by research from basketball, which has found that lower working memory capacity is related to how much a player will be distracted by irrelevant information during a match (Ashford et al., 2021). In soccer it has been shown that auditory distractions impact performance negatively for both elite players and novices on a tactical decision-making task (Glavas et al., 2023), and that the beneficial effects of prior contextual information on decision-making is lessened when a distractor-task must be performed simultaneously (Gredin et al., 2020). Together, this line of findings indicates that the degree of load on working memory is important for soccer performance.

Some research supports a “circumvention of limits”-hypothesis, which suggests that experts in a field can bypass the normal capacity limitations of working memory by relying on other memory systems to perform tasks quicker and more efficiently (Glavas et al., 2023). Specifically, some argue, extensive experience in soccer facilitates a separate domain-specific long-term-working-memory system (LT-WM) that can quickly retrieve and apply game-relevant information and patterns from long-term memory with no additional load to (short-term) working memory (Ericsson and Kintsch, 1995). This process may be closely related to pattern recognition skills (see Section 3.1.2). Support for LT-WM in soccer comes from studies showing that skilled soccer players generate more relevant choice options in computerized soccer decision tasks than less skilled players (Belling et al., 2015), can recognize and classify game situations quicker and more accurately than their less skilled counterparts (Lex et al., 2015), and can recall information from past matches with greater accuracy (Ashford et al., 2021). Further, a series of studies conducted by Roca et al. (2021, 2013, 2011) show a tendency for more skilled players to apply more advanced memory representations to soccer-related problem solving. Together, these findings support the assumption that elite soccer players have developed a domain-specific executive system, which can quickly retrieve and apply game-relevant information from long-term memory, as proposed by LT-WM theory. In further support of this, studies investigating the effect of non-conscious priming have revealed that quickly showing a soccer-related picture, which is later shown again to prompt a decision, can lower the response time for that decision for soccer players but not for non-players (Zoudji and Thon, 2003). Further, in an experiment with a shorter interval between prime and decision, a priming effect was shown for both novices and experts, but as working memory was overloaded with a secondary task, the priming-effect remained only for experts (Zoudji et al., 2010). These findings indicate that elite soccer players can efficiently utilize a separate memory system when information has not been registered consciously or when working memory is overloaded. However, while Glavas et al. (2023) did find that working memory capacity predicted speed and accuracy on a computerized soccer decision-making task, there was no interaction between working memory capacity and level of expertise. This indicates that experts and novices relied on working memory to a similar degree when solving problems. This finding goes against LT-WM-theory, which would predict that experts rely less on working memory because of their separate soccer-specific memory system. A systematic review on the factors distinguishing expert and novice performance in the integration of perceptual information by Brams et al. (2019) also finds more support for other theories than LT-WM. Rather than more efficient encoding and retrieval of visual information, they primarily found that expert performance was characterized by superior attentional allocation and perceptual ability.

Overall, the evidence supports the importance of working memory functions in soccer: When working memory is overloaded, performance tends to suffer. Also, more skilled players tend to perform better on working memory tasks, though results may vary depending on how broad a measure of working memory is used. Some studies used very broad measures of working memory function such as the design fluency test (Vestberg et al., 2020; Vestberg et al., 2012), which is also a measure of several executive functions. This makes it difficult to determine to which extent findings should be ascribed to working memory or other related concepts such as cognitive flexibility and control (see also Section 3.2.2). Further, some evidence suggests that highly experienced soccer players redelegate information normally processed by working memory to a separate domain-specific memory system, but the evidence for this is mixed, and it seems that elite players still rely on working memory to a significant extent. Finally, it is difficult to empirically disentangle the involvement of working memory in guiding perception and attention (Stage 1) from its role in influencing response selection (Stage 2). Since most studies simply measure the effect of working memory on the final decision outcome, it cannot readily be determined at which points in the cognitive processing it contributes.

### 3.2 Stage 2: action selection and execution

#### 3.2.1 Activation and selection between response options

Based on the assessment of the current situation from Stage 1, the player must decide which action to make. Our model conceptualizes the decision making process as a competition between response options, where the starting point is a comparison of the current play situation to representations of similar situations in long-term memory. Through extensive experience as a soccer player, certain actions have been linked to positive or negative outcomes in particular situations. For the elite player the situational assessment is highly specific, implying that very few actions (and sometimes only one) are associated to it. Neural populations in the brain’s motor systems related to each of these actions are activated at the beginning of Stage 2, but as only one action can be carried out, the actions compete for selection. This competitive process essentially constitutes an implicit evaluation of response options that results in the selection and execution of an action. There are two main determinants of the evaluation: First, how closely the present situation matches similar patterns in long-term memory. This matching also includes the player’s current proprioceptive state, that is, the readiness to perform certain actions. Second, how strongly the different actions have been linked to these patterns (the player’s response biases). By integrating these two factors, the selection process can combine present information with previous experience to maximize the probability for a successful outcome of the chosen action. The selected action also implies an expectation of its likely outcome, based on previous learning, which feeds into Stage 3 (i.e., assessment of outcome and feedback-based learning; see Section 3.3).

We assume that for elite soccer players, the action selection process occurs largely at an unconscious level, since decisions must be made fast and intuitively to take advantage of dynamic opportunities as they occur during the game. This is a different process in amateur players, where conscious deliberation seems to be more prominent (Starkes et al., 2004). The assumption is supported by research on both soccer and other team sports, which shows that a greater level of conscious processing with more deliberation time typically reduces the quality of decisions (Ashford et al., 2021; Fawver and Janelle, 2019). Whereas conscious thought is restricted by the capacity limitations of working memory, unconscious processing can evaluate several choice options simultaneously, and in any given situation during a soccer match, multiple response options are presumably relevant. It is highly unlikely that each option is sequentially considered and weighed against the others within consciousness, as this would take far too long (Petiot et al., 2021). Some studies have used verbal reporting to reveal superior recall and more sophisticated usage of memory by expert players in the context of soccer-related decision making (Roca et al., 2021, 2011). Such findings seem to indicate the use of conscious processing during action selection. The validity of these findings can however be questioned, since video-based tests without time restraints are probably quite dissimilar to real soccer matches and their fast coupling between perception and action (Zhao et al., 2022). Also, participants could retrospectively construct probable explanations for their own decisions. Instead, decisions may be “pre-reflected” in the sense that players are conscious of the decision itself but not the exact processes that gave rise to it Petiot et al. (2021).

The action selection processes are much harder to access empirically than Stage 1 processes. In previous sections we have described how more skilled players generally make quicker, better, and more creative decisions in soccer related tasks (Belling et al., 2015; Hüttermann et al., 2019; Wirth et al., 2018). Additionally, we have shown how proficiency in Stage 1 processes can predict superior decision making (Ashford et al., 2021; Belling et al., 2015; Glavas et al., 2023; Hüttermann et al., 2019; Lex et al., 2015; Natsuhara et al., 2020; Roca et al., 2018), and how changes in stimuli will impact decision making (Causer et al., 2013). However, whereas we can observe visual exploration behavior or measure working memory capacity with standardized tests and correlate these results with a decision making outcome, a player’s decision making proficiency *per se* is hard to measure: Since the response selection is based on the situational assessment it is difficult to determine if poor decision making is due to poor response evaluation, or if the evaluation was accurate but based on a poor situational assessment. Some studies have attempted to measure the evaluation process through retrospective verbal reporting of participants’ thinking during decision making (Roca et al., 2021, 2011). However, if the evaluation process for elite players is largely unconscious, verbal reporting of conscious thinking is not representative for on-field decision making. Because of the difficulties in measuring unconscious action selection, the literature on decision making in soccer is lacking in this important respect, and we therefore turn to general research on motor-related decision making.

Our modeling of the response selection processes at Stage 2 relates closely to the affordance competition hypothesis by Cisek (2007). This theory suggests that motor decision making depends on a competition between populations of neurons representing different movement options in a fronto-parietal system that integrates sensory, memory, and motor related information. As the current situation is perceived, it triggers response options based on past experiences of similar scenarios. The relevant response options compete, and while neurons within populations representing the same action will excite each other, neurons from different populations tend to inhibit each other. Eventually one neural population reaches a critical activation threshold, outcompetes the others, and the associated action is executed. Structures in the basal ganglia may function as gatekeepers for response selection in this process (Kalivas and Nakamura, 1999). The competition is resolved based on an ongoing analysis of costs, risks, and rewards (Cisek and Kalaska, 2010). The affordance competition hypothesis presents several explanations for decisional behaviors observed in soccer. As mentioned above, less familiar scenarios and fewer available good solutions tend to produce slower decision making (Ashford et al., 2021). Additionally, reaction times on decision tasks generally increase with the number of relevant options available (Czyż, 2021). The first finding can be explained neurophysiologically by the relatively lower activation in neuronal populations when the match between a current situation and stored representations is poor due to limited previous exposure to such scenarios. The second finding can be explained neurophysiologically by the fiercer competition between neural populations that will inhibit each other when no obvious decision presents itself. In both cases it will take longer for a neural population to reach the activation threshold, resulting in the observed slower response. Further, these neurophysiological considerations may help explain the “take-the-first” heuristic, which is prominent within team sports like soccer and suggests that a skilled player will typically pick the first viable response option that presents itself (Petiot et al., 2021). This heuristic could simply reflect behavior in scenarios that have become so familiar to players that response evaluation entails almost no competition, and a highly practiced motor response can be initiated almost immediately.

Another central point of the affordance competition hypothesis is that decision making is a continuous process representing an ongoing interaction between environment and actor (Cisek, 2007). Much research on decision making in soccer presents participants with a limited number of decision options at the same time, usually without requiring a complex motor response and with quite a lenient timeframe for response. However, decision making on the soccer field may correspond more closely to what can be termed embodied decision settings, where the environment is constantly changing and affords new response options in a way that blurs the lines between perception, decision, and execution (Gordon et al., 2021). From an evolutionary perspective, animals generally benefit from being able to adjust chosen decisions as new information comes online. Indeed, studies suggest that individuals executing a motor response will sometimes suddenly change their minds and switch course mid-action, when new information presents itself and makes another response more beneficial (Cos et al., 2021). This highlights that both situational assessment and action selection continues well into motor execution, which includes the preliminary processes in the body and muscular system associated with this. Additionally, neuroimaging research generally supports the notion of an integrated anatomical network of perceptual, knowledge-based and motor-related areas where the same neurons are continually involved in all processes (Cisek and Kalaska, 2010). For our model, this means that action selection is continuously on-going and that each processing stage is updated as new perceptual and cognitive information becomes available. The situational assessment from Stage 1 will continue to update and bias response selection in Stage 2, and even after motor execution has begun, a new decision may reach the activation threshold and change motor behavior in another direction. Conversely, difficulties in reaching a decision at Stage 2 may initiate more explorative behavior at Stage 1 to provide sufficient information for the decision, so the two stages function in an interactive manner. Here, our model differs from classical tenets of information processing, which assumes decision making to be a sequential procedure of functionally and neuroanatomically distinct processes that are each completed before the next can begin: first perception, then decision, then execution (Cisek and Kalaska, 2010).

Though the specific details of motor control processes are very relevant for soccer in general, and as targets for systematic training, they do not fall within the explanatory scope of our cognitive model. However an important general point is that motor competence, the ability to execute a particular movement pattern, is a precondition for action selection. For example, a bicycle kick requires very specific motor skills and is not part of the action repertoire of most amateur soccer players. This implies that the neural and bodily activity corresponding to a bicycle kick is not activated during Stage 2 for such players. However, given appropriate physical fitness, this may be altered by training of this particular motor skill, in which case the option of a bicycle kick can enter Stage 2’s action selection in relevant play situations. Another way of describing this is that a motor representation of a bicycle kick now exists in the player’s procedural long-term memory, from which it can be activated by relevant situational cues perceived at Stage 1.

In this section we have argued for Stage 2 as a competition between response options, in which the activated options are evaluated based on a match between the situational assessment, stored situational representations, and the strength of associations between these representations and specific actions. While there is limited research on the processes involved in action selection specifically for soccer, mainly due to their unconscious and elusive nature, research from the general field of motor decision making supports the idea of a competitive decision making process. The affordance competition hypothesis offers a theoretical framework for this process, including a description of action selection as a continuous process that regulates decisions all the way until they are carried out.

#### 3.2.2 Executive functions

Whereas the action selection process itself is essentially non-conscious, its outcome can be influenced by conscious intentions and other cognitive control processes. This adds flexibility to an otherwise automatically driven behavior, which presumably is very important in a dynamic and complex game like soccer. Conscious control also leaves room for coordination between players in the form of team strategies and instructions from the coach. On the other hand, as discussed in the previous section, there seems to be a trade-off between the flexibility offered by cognitive control and the speed gained by automatic reactions. Cognitive control can take many different forms, which fall under the general heading of executive functions. Executive functions are typically divided into core and higher-level processes. Core executive functions are relatively basic control processes that belong to one of three main categories: mental shifting, information updating, and inhibitory control (Diamond, 2013; Friedman and Miyake, 2017; Miyake et al., 2000). Information updating is closely related to working memory function as it was described in relation to Stage 1 (see section 3.1.4). Higher-level executive functions combine several core processes for handling of complex tasks, for example in relation to planning, reasoning, and creative problem solving. There is some general evidence that executive functions are important in sports. For example, Jacobson and Mattheus (Jacobson and Matthaeus, 2014) found that athletes tend to outperform non-athletes on tests of inhibition and problem solving, and Rahimi et al. (2022) found that athletes in strategic sports (such as soccer) scored higher on response conflict tasks than athletes in non-strategic sports (such as track-and-field). Executive control functions are also relevant to processes at Stage 1, especially orienting behavior, but as mentioned in Section 3.1.1 this is not directly addressed in the empirical literature.

In soccer research executive functions are typically investigated by standard cognitive tests such as Trail Making B (mental shifting), Stop-Signal and Stroop tasks (response inhibition), and Design Fluency (higher-level executive processes). Several studies have reported that skilled soccer players tend to score better than average on such cognitive tests, and that the executive scores correlate with actual play performance. Vestberg et al. (2012) studied two groups of players: Swedish national league players (High-division; HD) and players of lower divisions (LD). They compared scores on the Design Fluency test between these groups, as well as a standardized norm group. Both the HD and LD players scored much better than the norm group, and the HD players also scored significantly better than their LD counterparts. Furthermore, a significant positive correlation was found between the Design Fluency score and the number of goals and assists two seasons later. Vestberg et al. (2020) followed up by showing that Swedish national team players scored significantly higher on the Design Fluency test than lower-ranking professional players (as well as much better than the norm group). The effect was specifically driven by the third Design Fluency subtest, which is the most cognitively complex part of the task. In addition, this test score correlated significantly with coach-rated game intelligence as well as with the number of assists (but not goals) made during a season. These findings support the notion that high-level executive functions are related to the ability to adaptively “read the game” that is highly valued in soccer.

Another topic that is related to flexible action selection in soccer is creativity. Creativity can be defined as the ability to produce solutions that are both novel and appropriate across different situational contexts (Roca et al., 2018). In soccer, creative tactical actions are typically more surprising to opponents than conventional playing and can provide an important competitive advantage. Indeed, Kempe and Memmert (2018) found that national teams exhibiting a higher degree of creativity in their goal scorings progressed further in the FIFA World Cup tournaments in 2010 and 2014. As mentioned in Section 3.1.1, creativity in soccer seems related to a higher degree of visual exploration (Roca et al., 2018). This is in line with our model’s assumption that flexible response generation at Stage 2 is related to more elaborate information gathering processes at Stage 1, which tend to activate more than a single response option. Knöllner et al. (2022) also found significant correlations between tests of executive and visual functions in elite soccer players, further pointing to connections between these two cognitive domains in soccer. Presumably, the level of creativity in soccer players is also related to training and match-playing policies in different clubs, but to our knowledge this has not yet been systematically investigated. Higher levels of creativity could result both from conscious choices of playing attitude as well as automatic, overlearned processes established by training regimes that emphasize response flexibility.

In summary, executive functions can be assumed to be highly relevant for skilled soccer performance, mainly due to the response flexibility they provide. In line with this general theoretical point, there is some empirical evidence that performance on higher-level executive tests is positively related to match performance. Creativity is an important aspect of response flexibility, and seems related to more elaborate perceptual exploration at Stage 1. However, the empirical evidence on executive functions in soccer is still quite limited. Methodological concerns have also been raised about the reliability and validity of executive tests, which were typically developed for clinical purposes, in the different context of sport research (Furley et al., 2023). For example test impurity may be problematic, as executive functions to a large degree work through other cognitive functions, which complicates the interpretation of results. A related point is that studies using soccer-specific tasks and naturalistic research designs are still lacking in this research field.

### 3.3 Stage 3: assessment of outcome and feedback-based learning

#### 3.3.1 Behavioral learning and reinforcement

When the action has been fully carried out the player perceives the outcome. In our model this stage completes the perception-action cycle by modifying cognitive and neural settings for future situations. This way Stage 3 provides a crucial learning element to the whole process. The outcome is perceived as either successful or unsuccessful relative to the intention for the action, but to a varying degree. Scoring a goal at a decisive moment in the most important match of the year probably represents one of the highest levels of perceived success, whereas a simple pass in a low-stakes situation implies a more moderate outcome evaluation. In any case, the assessment of the action outcome is subjective in nature and not necessarily equivalent to the objective costs or benefits for the team. Instead it relates to the player’s personal goals and level of self-interest, which may overlap with the team, but can also – for example in case of social competition between teammates - be contrary to the collective aim. The learning impact of the assessment is closely coupled with the player’s expectations for the outcome, which is an inherent aspect of the action produced in Stage 2: An action with a low expectation of success, such as a long shot at the goal, will lead to stronger behavioral feedback if successful than an action which the player expects to carry out well every time. Vice versa, failure of a normally successful action leads to stronger behavioral feedback than failure of an action with a low expected probability of success.

This general principle relates to the theory of reward prediction error, which states that the difference between predicted and received rewards is the central mechanism for behavioral learning (Glimcher, 2011). At the neurophysiological level prediction errors are indexed through up- and downregulation of activity in dopaminergic neurons, which signal the amount of deviation from the predicted outcome (Schultz, 1998). Given the widespread projections of the dopaminergic system to anterior parts of the brain, this activity can influence many cognitive processes, in particular those related to action selection. In our model this corresponds to plastic changes in the relative balance between excitation and inhibition of specific responses at Stage 2. The dopaminergic activity also feeds back to the orientation movements at Stage 1 that immediately preceded the action, and reinforces this behavior either positively or negatively. This way the assessment of the action outcome makes the player either more or less likely to repeat the associated behavior in similar future situations.

Soccer-related studies of these learning processes are still in their infancy, but Häusler et al. (2015) published an fMRI study that showed overlap in mesolimbic dopaminergic activity between monetary and social (soccer-related) rewards. This indicates that the general neural mechanisms of reward learning also apply in the context of soccer. In addition, Häusler et al. (2015) found an interaction with egoism personality scores, so that activation of the left middle frontal gyrus when scoring indirectly via a pass versus by a direct shot correlated with this personality measure. These are interesting pioneer findings, but clearly much more research is needed to bridge the empirical gap between general theories of behavioral learning and soccer-specific processes. At present, our case for the relevance of the reward prediction error theory in soccer rests on its status as a highly established general theory, not on direct evidence in the field of soccer research.

#### 3.3.2 Attentional reorienting, perceptual learning, and metacognition

The outcome assessment has other effects on the player’s cognitive processes than modification of automatic action tendencies. These other effects relate both to the attentional and perceptual processes at Stage 1 as well as the executive control over response processes at Stage 2. As described in the previous section the dopaminergic feedback mechanisms also influence orientation behavior at Stage 1. Given the close coupling between exploratory behavior and attentional focus (Rizzolatti and Luppino, 2001) these behavioral modifications imply changes in attentional processes, for example a stronger tendency to monitor a particular spatial direction if something unexpected just occurred from there. However the outcome assessment also has effects on purely perceptual processes, especially relating to pattern recognition and anticipation. The ability to recognize and predict dynamic play configurations in soccer can be assumed to depend on the accumulated experience from many thousands of individual situations (Ericsson et al., 1993; Williams et al., 2012). Every time a player perceives the outcome of a play situation, a small modification is made to these highly specialized perceptual abilities. The long-term result of this learning process is the general advantage that elite players hold over less skilled players in terms of pattern recognition and anticipation in soccer, as described in Sections 3.1.2 and 3.1.3.

The executive control processes at Stage 2 are also influenced by the outcome assessment. In particular, the risk taking aspect of response selection can be assumed to depend on the current confidence level of the player. Feelings of confidence are closely related to metacognitive processes, where metacognition can be defined as the ability to monitor the accuracy of one’s own cognitive and behavioral functions (Fleming, 2024; Fleming and Frith, 2014). The confidence level for a given ability is dynamically modified by individual experiences when performing a given activity (Rouault et al., 2019). This way, the outcome assessment can provide the necessary feedback to continuously modify (positively or negatively) the player’s feeling of confidence, and thereby the tendencies to select particular actions.

## 4 General discussion

In this paper we have presented a three-stage model of the cognitive processes involved in each play situation of soccer at the elite level. The model provides an integrating theoretical frame for how cognitive processes interact during high-level soccer play. It includes the main cognitive processes that have been studied empirically in soccer research, but also points to a number of important processes that are as yet little investigated. In the following we discuss the empirical and theoretical basis of the model, its applied perspectives, and implications for future research.

As detailed in the review section of this paper, the model relates directly to specific fields of investigation in cognitive soccer research. The model can accommodate the wide variety of findings in this empirical literature, and it provides a general organizing frame for cognitive soccer research. However our review also points to several important limitations in the current evidence. First, a large part of the existing research is laboratory-based, but cognition and behavior in these settings does not necessarily translate to real-life play. Indeed, several studies point to important differences between performance in laboratory and real-life settings, for example in relation to gaze patterns. For this reason there is a growing acknowledgement that studies should use research designs and tasks that are more representative of real play situations, and compare cognitive measures directly to match performance. While the methodology for such naturalistic soccer studies has been developing rapidly in the last few years, there is still limited evidence to bridge the gap between measures of cognition in laboratory environments and actual match performance. Second, the large majority of situations for players during a soccer match do not involve ball possession, but rather positioning for defensive or attacking purposes. However, studies of cognitive processes associated with positioning behavior are virtually absent in the empirical literature. Third, even under controlled experimental conditions, it remains difficult to empirically disentangle the different cognitive processes that contribute to soccer behavior. This is especially the case for processes that are involved at multiple processing stages, such as working memory and executive functions, and for the unconsciously operating mechanisms of action selection. Fourth, there is sparse evidence on many details of the processes included in our model, for example soccer-specific pattern recognition, involvement of other sensory systems than vision, individual differences in risk taking during response selection, or the different learning mechanisms of Stage 3. This way, even though much progress has been made in cognitive soccer research over the last two decades, the ecological validity of many studies can be questioned, and important questions remain unanswered. This implies that many aspects of our model await further validation.

Partly due to these limitations of the soccer literature, our model relies significantly on general theory and evidence from the wider research fields of cognitive psychology and neuroscience. For Stage 2 we apply general principles from the affordance competition hypothesis to describe action selection in soccer. This well-established theory of motor-related decision making should be highly relevant to soccer: both the speed and sensory-motor nature of soccer makes an embodied decision making approach more appropriate than traditional cognitive models of decision making, which rely heavily on (slow) conscious deliberation. The affordance competition hypothesis describes a parallel competition process between action programs, which might seem at odds with the overall serial format of our model. However, as emphasized several times in the paper, our model includes parallel and interactive processes both within and between the three main processing stages. The affordance competition hypothesis is therefore fully compatible with the other elements of the model. Still, our account of Stage 2 rests mainly on general theoretical considerations rather than direct evidence on how actions are selected by soccer players. The same is the case for the feedback processes of Stage 3, where we apply general principles of cognitive and neural learning from the reward prediction error theory and other research. Also with regard to these learning processes, soccer-specific empirical studies are much lacking. While it is reasonable to assume that decision-making and learning processes in soccer follow the same principles as human cognition and behavior more generally, the lack of direct evidence is a significant limitation of our model, which will have to be addressed by future studies.

Professional soccer is performed in a high-pressure environment, and an important question is how emotions and stress-related factors influence the cognitive processes described in the model. Emotions can have both positive and negative effects on sport performance (Vast et al., 2010) and are likely to influence many different cognitive functions (Smith and Lane, 2015). For this reason emotions or stress-related processes are not associated with specific parts of our model, but should rather be considered as general modifying factors across the whole perception-action cycle. That said, some cognitive processes are probably more vulnerable to stress and intense emotionality than others. It is often suggested that relatively attention-demanding processes, in particular executive control functions, are more susceptible to emotional influences [for a recent study of this in soccer players, see Knöbel et al. (2024)]. Conversely, automatic processes like pattern recognition and highly overlearned responses are typically more robust to emotional pressure. Attention-dependent processes are also related to arousal and cognitive effort (Kahneman, 1973), both of which can be influenced by stress and emotionality. In a soccer context these processes have mainly been studied in relation to mental fatigue [see Soylu et al. (2022) and González-Víllora et al. (2022) for recent reviews of this emerging research field]. Also, following the classical work of Kahneman (1973), changes in pupillary size may function as a marker for cognitive effort in soccer (Cardoso et al., 2019). Clearly, much interesting research awaits to be done on the interaction between emotional arousal and cognition in soccer.

Due to its cognitive specificity and coverage of the whole perception-action cycle our model has important applied perspectives, especially in relation to professional soccer training. For example the model allows for a detailed analysis of the cognitive processes involved at Stage 1, which can potentially widen the focus of training to include pre-decision processes rather than the currently dominating focus on decision making and situational outcomes. As we argued in Section 3.2.1, a decision to perform a certain action can only be as good as the preceding situational assessment allows, so it is very important to optimize Stage 1 processes. From an analytic perspective the advantage of such an approach is also that, whereas the outcome of a given tactical situation depends on many external factors, a player’s cognitive preparation before making an action can be analyzed more specifically. Visual orientation behavior is for example readily observable by video analysis. We would argue that such observational studies could be performed by the analysis department possessed by most elite soccer clubs, and that these data could be supplemented by standardized testing of specific cognitive abilities for each player. Such combined analyses could give a more complete and individualized understanding of the decision making processes of elite soccer players, and provide a basis for training specific cognitive aspects thereof. This cognitively focused training could be organized in a periodization structure or via individual development plans for each player. The details of such periodization structures and training methodologies are however beyond the scope of the present article.

Based on our theoretical model and review of the empirical literature we would like to conclude this paper with some general suggestions for future research. To address one of the most important limitations of the existing research on cognition in soccer, it is important to expand the recent trend toward more naturalistic studies. Specifically, in the pursuit of experimental tasks and measures that are more representative of actual soccer play, studies that focus on positioning behavior rather than ball possession would be very informative. The influence of emotional factors on cognition in soccer is another underdeveloped research area with huge potential relevance for professional performance. Finally, important scientific advancements could be gained from research designs that include cognitive interventions. Contrary to the correlational designs that have been used in most soccer studies so far, experimental interventions that systematically manipulate cognitive variables can address mechanisms of causality more directly. Such research designs are also better suited to disentangle the contribution of different cognitive processes to behavioral outcomes, which is currently a major limitation for interpreting the evidence. If the cognitive interventions represent activities that can transfer to professional soccer training, such studies would also have large applied perspectives.

## Data Availability

The original contributions presented in the study are included in the article/supplementary material, further inquiries can be directed to the corresponding author/s.
